# Successful use of pulsatile flow and goal directed perfusion in a high-risk patient

**DOI:** 10.1051/ject/2025002

**Published:** 2025-09-15

**Authors:** Mostafa Bagherinasab, Nathaniel H. Darban

**Affiliations:** 1 Extracorporeal life support, Baqiyatallah University of Medical Sciences Sheikh Bahayi St. Mollasadra St. Vanak Square Tehran Iran; 2 Midwestern University 19555 N. 59th Avenue Glendale Arizona 85308 USA

**Keywords:** Oxygen delivery index, Cardiac index, Pulsatile flow, Organ perfusion

## Abstract

The development of multi-organ failure resulting from cardiopulmonary bypass (CPB) is acknowledged as a significant contributor to increased morbidity and mortality rates during the postoperative period. This report discusses a patient who presents with multiple comorbidities, including renal failure, reduced ejection fraction, and a history of hypertension, and is being considered for coronary artery bypass grafting (CABG) along with aortic valve replacement surgery. The administration of CPB was customized to address the unique comorbid conditions of the patient, highlighting the critical objective of maintaining an oxygen delivery index (DO_2_i) exceeding 280 mL/min/m^2^, while also integrating pulsatile flow methodologies. The management of CPB, as previously discussed, resulted in a notable enhancement of kidney function, accompanied by a reduction in the patient’s lactate levels post-surgery.

## Overview

Open-heart surgeries represent intricate medical interventions that entail significant risks and are linked to a wide array of possible complications after the surgery [1]. The occurrence of multi-organ failure as a consequence of CPB is recognized as a prevalent factor contributing to both morbidity and mortality in the postoperative period [2]. The idea that the modulation of pump flow should be aligned with the DO_2_i rather than depending exclusively on body surface area (BSA) and a pulse index (PI) between 1.8 and 2.4 L/min/m^2^ is a widely debated subject among researchers [1, 3]. Considerable efforts have been focused on discovering methods to safeguard organs, and among these initiatives, pulsatile flow (PF) and modifications in blood flow during CPB have been recognized as important factors.

This report examines the utilization of a synergistic approach that incorporates PF alongside an enhanced CI to evaluate organ perfusion throughout the process of CPB.

## Description

A 70-year-old male, a former smoker, with hypertension, and dyslipidemia presented with symptoms of dyspnea and angina. The patient required hemodialysis a decade prior to the surgical procedure, which was necessitated by a significantly reduced ejection fraction (EF) of 35%. Cardiac catheterization revealed triple vessel coronary artery disease and severe aortic valve insufficiency, and he was scheduled to undergo CABG and aortic valve replacement. In light of the patient’s medical background, which includes renal failure requiring hemodialysis, advanced age, anemia, hypertension, and the expected extended duration of CPB along with aortic clamping, the perfusions’ opted to implement pulsatile flow alongside an increased CI to ensure sufficient perfusion of essential organs.

The individual presents with a height of 174 cm, a weight of 84 kg, a BSA of 2.01 m^2^, a PI of 2.6 L/min/m^2^, and a flow rate of 5220 mL/min/m^2^, calculated using the Du Bois & Du Bois formula. The CPB system included an Inspire 8F oxygenator (LivaNova, Mirandola, Italy) and an Inspire hard-shell venous reservoir (LivaNova). The equipment comprised of a Stöckert S5 heart–lung machine with roller pump (LivaNova) and a Stöckert Heater Cooler System 3T (LivaNova). Due to a hemoglobin level of 9 g/dL before the commencement of CPB, a priming solution consisting of 1 L of Ringer’s solution along with 350 mL of packed red blood cells (PRBC).

Additionally, 200 mL of mannitol and albumin were included in the CPB prime. After administering heparin at a dosage of 400 IU/kg, the patient was subjected to cannulation of the aortic root, which was followed by the placement of venous bicaval cannulation. Throughout the CPB, the activated clotting time was sustained at a level greater than 480 s. The patient experienced controlled mild hypothermia during CPB, achieving a body temperature of 33 °C. The administration of 1250 mL of Del Nido cardioplegia (DN) was performed, which comprised a combination of one-part oxygenated blood mixed with four parts of the DN crystalloid solution. The administration of a half-main dose of DN cardioplegia was repeated at intervals of every 90 min.

PF was established after aortic cross clamp and cardioplegia injection, characterized by a base flow of 30%, a width of 60%, and a frequency adjusted to 70 beats per minute. Quality of the pulsatile flow was assessed using Energy Equivalent Pressure (EEP). Modifications to the width and frequency settings were implemented to ensure that the EEP exceeded the Mean Arterial Pressure (MAP) by 15% [4]. Furthermore, it is important to note that in the recent quantification of pulsatile flow, the EEP is utilized to evaluate the quality of pulsatile flow by calculating the pressure beneath the arterial pressure and the CPB flow curve during the pulsatile cycle. In this context, it is essential to maintain the EEP value at 10–15 mmHg above the MAP [4].

Throughout the course of CPB, a specific strategy was employed to ensure that hemoglobin concentrations remained above 8 g/dL. This approach included the transfusion of three units of PRBC in conjunction with a three-liter hemofiltration process. In this case, the patient encountered volume overload due to renal failure, dysfunction of the aortic valve, and a diminished EF. As a result, there was a considerable need for improved hemoconcentration to effectively manage these complications. In instances of hypotension, we elevated the flow rate to 6 L/min/m^2^, corresponding to a PI of 3 L/min/m^2^. However, if the hypotension persisted with a MAP below 60 mmHg, we administered norepinephrine as a bolus dose of 20 µg/cm³. Throughout the course of cardiopulmonary bypass, a total of 10 mg of norepinephrine was utilized.

The average DO_2_i ranged from 280 to 300 mL/min/m^2^. The calculation of DO_2_i was performed using the following formula: DO_2_i (mL/min/m^2^) = pump flow (L/min) × [Hct/2.94 (g/dL) × 1.36 × arterial oxygen saturation (%) + partial pressure of arterial oxygen (mm Hg) × 0.003] × 10/BSA (m^2^). The B-Capta online blood gas monitoring system developed by LivaNova was employed, facilitating precise measurements of partial pressure of oxygen (pO_2_) and temperature within the arterial line, as well as saturation, hematocrit (HCT), hemoglobin (Hb), and temperature in the venous line. The calculation of DO_2_i was performed manually at 15-minute intervals, utilizing the specified formula.

Following an aortic clamp duration of 112 min and a CPB period of 130 min, the patient was successfully weaned from the heart-lung machine (HLM) without the need for any mechanical assistance or pharmacological support.

An outstanding feature of this case was the enhancement of the EF to 45%, which ultimately removed the requirement for hemodialysis after the surgery, as well as a reduction in lactate levels after CPB when compared to pre-operative measurements. The fluctuations in creatinine and lactate levels are depicted in Tables 1 and 2.

**Table 1:** Creatinine variations.

Parameter	Base	After surgery	12 h after surgery	24 h after surgery	48 h after surgery	72 h after surgery
Creatinine (mg/dL)	4.8	4.8	4.6	4.6	3.8	2.6

**Table 2:** Lactate level fluctuations.

Variable	Base	30 min after CPB initiation	60 min after CPB initiation	90 min after CPB initiation	120 min after CPB initiation	ICU admission	6 h after CPB weaning	12 h after CPB weaning	24 h after CPB weaning
Lactate level (mmol/L)	2.4	2	1.8	2	1.4	2.1	1.4	1.2	0.9

## Comment

The limitations related to lactate as a robust predictive marker are grounded in the understanding that hyperlactatemia may arise from a combination of factors occurring both during and after surgery [5]. The association between diminished flow rates (below 100 mL/kg/min) and DO_2_i during CPB, in conjunction with the duration of CPB and circulatory arrest, HCT levels, temperature variations during and post-CPB, as well as systemic inflammatory responses, has been shown to have a significant correlation with both intraoperative and postoperative lactate concentrations [6, 7].

The capacity of DO_2_i to accurately forecast sufficient organ perfusion, especially within the renal system, has been evidenced by a considerable sample size, with a specific DO_2_i threshold established at 270 mL/min/m^2^ [8]. In this case, we maintained the DO_2_i level above 280 mL/min/m^2^.

Within the context of this patient case, we implemented PF to enhance organ perfusion following an increase in PI. End-organ dysfunction and the duration of recovery following cardiac surgery are influenced by both non-CPB and CPB-related factors. Among the CPB-related factors, the activation of inflammatory and coagulation pathways resulting from blood exposure to synthetic materials, the absence of natural pulsatile flow, ischemic damage, embolic events, and hypothermia are significant contributors. Notably, the absence of physiological pulsation associated with non-pulsatile CPB is regarded as a critical factor leading to end-organ dysfunction and an extended recovery period post-surgery [9–11]. The conceptual benefits of pulsatile CPB over its non-pulsatile counterpart primarily arise from its capacity to mimic the body’s inherent hemodynamic environment. This mimicry produces more physiologically pertinent pulsatile waveforms, thereby promoting improved mechanical energy transfer to the vascular endothelium [12, 13]. The cyclic endothelial shear stress that results from this process promotes the enhanced secretion of vasodilatory agents, which in turn reduces vascular resistance and improves perfusion and microcirculation in the target organs [14, 15].

The improvement in left ventricular ejection fraction (LVEF) observed in this instance can be attributed to the revascularization of coronary arteries, alongside the initiation of certain pharmacological agents, including beta-blockers. These medications have the potential to enhance cardiac function by facilitating adjustments in both preload and afterload conditions [16].

A higher PI during CPB is associated with improved LVEF after CABG because it ensures sufficient myocardial perfusion and oxygen delivery, thus reducing ischemic damage to the heart muscle [5]. Coupling this with pulsatile flow can further enhance these benefits; pulsatile flow mimics natural physiological conditions, promoting better hemodynamic stability and improving microcirculation to vital organs, including the myocardium [17]. Together, these interventions can significantly contribute to better myocardial function and recovery and subsequently enhanced postoperative LVEF.

It has been previously noted that we performed three liters of hemofiltration in response to volume overload [18]. This raises the consideration that research suggests the intensive application of hemofiltration during CPB can result in either absolute or relative hypovolemia, potentially jeopardizing renal perfusion. Sufficient kidney perfusion is indicated by the presence of PF, which enhances the delivery of oxygen to the tissues. This is supported by a DO_2_i value exceeding 280 mL/min/m^2^, demonstrating the effectiveness of this perfusion in meeting the metabolic demands of the kidneys.

Research indicates that the physiological characteristics of arterial PF can be effectively replicated through the use of HLM pulsatile flow settings [19]. We suggest that the combination of an enhanced PI with the use of PF may lead to a gradual enhancement of organ perfusion during CPB.

This study offers an innovative approach to assessing organ perfusion by altering the PI, while simultaneously employing PF methodologies.

## Data Availability

The article integrates all accessible data into its content.
